# The association of dyslipidemia and obesity with glycated hemoglobin

**DOI:** 10.1186/s40842-015-0004-6

**Published:** 2015-06-23

**Authors:** Jayesh Sheth, Ankna Shah, Frenny Sheth, Sunil Trivedi, Nutan Nabar, Navneet Shah, Premal Thakor, Rama Vaidya

**Affiliations:** 1 Department of Biochemistry and Molecular Genetics, FRIGE’s Institute of Human Genetics, FRIGE House,, Jodhpur Gam Road, Satellite, Ahmedabad, 380015 India; 2 Unit of Endocrine and Metabolic Disorders, Kasturba Health Society, Medical Research Centre, Mumbai, 400056 India; 3 Department of Diabetes and Endocrinology, Sterling Hospital, Ahmedabad, 380052 India; 4 Gujarat Diabetic Association, Ahmedabad, 380007 India

**Keywords:** Dyslipidemia, Obesity, T2DM, HbA1c, non-HDL-C

## Abstract

**Background:**

Dyslipidemia and obesity are the most common complex metabolic disorders taking the highest toll of health resources globally by its increasing incidences. This consequently leads to type 2 diabetes mellitus (T2DM) and cardiovascular disorders (CVDs) with variable reports about the role of metabolic factors on glycemic control. The current study is designed to determine the association of dyslipidemia and obesity with glycated hemoglobin (HbA1c) in T2DM and non-diabetic subjects.

**Methods:**

The present study was carried out in 931 subjects from urban Western India including 430 diabetic and 501 non-diabetic subjects with detailed anthropometric parameters. All subjects were investigated for HbA1c and lipid parameters like TC, TG, HDL-C, LDL-C and non-HDL-C.

**Results:**

Dyslipidemia, central- and peripheral- obesity were observed (50.27 %; 75 % and 59.83 %) in all the study subjects respectively. Additionally, hyper-non-HDL-C was detected in 23.49 % and 22.56 % in T2DM and non-diabetic subjects. Significant linear associations of hyper-TC, hyper-LDL-C and hyper-non-HDL-C were observed with HbA1c in T2DM and non-diabetic control subjects respectively. Centrally- and peripherally- obese dyslipidemic subjects also showed a significant association with HbA1c in T2DM and control subjects respectively.

**Conclusion:**

This study demonstrates the high prevalence of dyslipidemia and obesity in all subjects irrespective of their disease status in a Western Indian population. The dyslipidemic obese subjects had significant linear association with HbA1c in T2DM subjects.

## Background

Dyslipidemia is associated with more than half of cases with ischemic heart disease and more than 4 million deaths per year [1]. In India, a rise in obesity and dyslipidemia with increasing urbanization have led to various lifestyle related disorders like T2DM, CVD and metabolic syndrome [1]. Among dyslipidemic subjects, increased LDL-C, TG and hypo-HDL are established markers for CAD risk. Additionally, TG-rich lipoproteins like VLDL and IDL contribute to total atherogenic cholesterol and are reflected by non-HDL-C which can be used as a secondary target for lipid lowering agents as proposed by the National Cholesterol Education Program, Adult Treatment Panel III (NCEP-ATP III) [2, 3] and may be used as the best risk predictor for stroke and cardiovascular complications [3].

Moreover, more than half a billion people worldwide are obese [4, 5] and has serious impact on multiple health outcomes [6]. World Health Organization (WHO) recognizes obesity and its complications amongst the top 10 global risk factors leading to ~40 % global deaths [7]. The menace of obesity is attaining an epidemic worldwide due to changes in life style and food habits. It is defined as a state of excess adipose tissue mass, and not by the body weight alone since muscular individuals may possibly have overall weight gain or high body mass index (BMI) without an increase in adiposity. Thus, those who are not peripherally obese or overweight might be centrally obese with high percentage of body fat distributed predominantly in the abdominal region [8]. In centrally obese individuals, adipose tissue releases higher amounts of non-esterified fatty acids (NEFA), glycerol, hormones, pro-inflammatory cytokines contributing to insulin resistance (IR) [9, 10]. Asian Indians are found to have higher intra-abdominal fat, visceral fat and metabolic disturbances as compared to Caucasians [8, 11] with 3 out of 4 (73 %) urban Indians being overweight [12]. Based on recently reported prevalence of obesity in India, it has been shown that every second urban Indian is obese with the highest risk of weight gain between 28–38 years [12].

These metabolic dysregulators like dyslipidemia and obesity along with other lifestyle factors are known to be associated with T2DM, [2, 13–15] characterized by hyperglycemia [16]. As per the International Diabetes Federation (IDF) 2013 estimate, India ranks second amongst the top 10 countries with 65.1 million diabetic subjects with an alarming high rate. It is further set to increase up to 109.0 million with a global estimate of 592 million by the year 2035 [17].

IDF estimates that nearly 175 million individuals are undiagnosed with diabetes globally [17]. The disease manifestation takes place much before its clinical appearance; subjects with T2DM are often asymptomatic initially as early as 12 years before the diagnosis and continue to remain asymptomatic throughout the disease process. Consequently, the age of diagnosis may not accurately reflect the age of onset of the disease [18].

It will be fruitful to detect early diabetics in India to curtail the ever increasing incidence of T2DM so that suitable lifestyle modifications may be suggested to prevent or postpone the onset of T2DM. Our study therefore attempted to demonstrate the influence of dyslipidemia and obesity on HbA1c in diabetic and non-diabetic subjects and whether can it be advocated as a combined ‘biomarker of lifestyle pattern’. Moreover, the novelty of the study is an attempt to demonstrate the association of combined effect of dyslipidemia and obesity on HbA1c in Western Indian urban population due to paucity of such studies [19].

## Methods

### Subjects

The current prospective study was carried out on the Western Indian population (Gujarat and Maharashtra) that comprised of 931 unrelated participants (430 previously diagnosed T2DM patients and 501 non-diabetic control subjects) in the age range of 25 to 89 years. They were enrolled by consecutive sampling during April, 2012 to October, 2014. All T2DM subjects satisfied the inclusion criteria of age ≥25 years, duration of diabetes (≥6 months from the date of diagnosis) and plasma glucose level [Fasting plasma glucose (FPG) >126.0 mg/dl, post prandial plasma glucose (PPPG) >190.0 mg/dl]. The inclusion criteria for control subjects were age ≥25 years, plasma glucose level (FPG) <110.0 mg/dl) and HbA1c level ≤6.5 %. The study was approved by the institutional ethical committee and ISBEC (Intersystem Biomedica Ethics Committee). Informed written consent from all participating subjects was obtained prior to the enrolment. Those with T1DM, other concomitant illness, lactating and pregnant mothers and those who were on medication of lipid lowering agents (statins, fibrates) were excluded from the study. Anthropometric parameters such as BMI, waist circumference (WC) and duration of T2DM were recorded for all the participating subjects as shown in Table 1. T2DM patients were treated with biguanides, sulphonylureas, PPARγ activators, DPP4 inhibitors and α glucosidase inhibitors alone or in combinations as advised by their physicians.

**Table 1:** Anthropometric and biochemical parameters in T2DM and control subjects

Parameters (T = 931 subjects)	T2DM (n = 430)	Control (n = 501)	*p* value
	Mean ± SD (range)	Mean ± SD (range)	
**Anthropometric indices**			
**Age (Yrs.)**	56.57 ± 10.52	48.60 ± 12.95	-
	(28.0-86.0)	(25.0-86.0)	
**BMI (Kg/m** ^**2**^ **)**	27.34 ± 5.12	25.83 ± 4.62	0.092
	(16.26-51.31)	(12.70-47.42)	
**WC (cm)**	96.88 ± 10.28	92.11 ± 10.72	0.542
	(65.0-142.0)	(64.0-128.0)	
**Biochemical Parameters**			
**FPG (mg/dl)**	140.79 ± 48.31	88.27 ± 12.39	<0.001^*^
**PPPG (mg/dl)**	189.53 ± 66.85	ND	-
**HbA1c (%)**	8.18 ± 1.81	5.68 ± 0.51	<0.001^*^
**FI (uIU/ml)**	11.02 ± 6.47	11.97 ± 5.44	0.487
**HOMA-IR**	3.85 ± 2.30	2.67 ± 1.56	<0.001^*^
**TC (mg/dl)**	181.56 ± 52.01	181.97 ± 46.18	0.008*
**TG (mg/dl)**	142.04 ± 73.30	112.03 ± 53.80	<0.001^*^
**HDL-C (mg/dl)**	54.24 ± 16.77	56.79 ± 17.33	0.151
**LDL-C (mg/dl)**	97.94 ± 50.33	102.13 ± 45.14	0.079
**Non-HDL-C (mg/dl)**	127.32 ± 53.21	125.18 ± 47.10	0.034*

By Independent Student’s *t*-test, *: Significant, SD: Standard deviation

BMI: Body mass index, WC: Waist circumference, FPG: Fasting plasma glucose, PPPG: Post prandial plasma glucose, HbA1c: Glycated hemoglobin, FI: Fasting Insulin, HOMA-IR: Homeostasis Model of Assessment-Insulin Resistance Index, TC: Total cholesterol, TG: Triglycerides, HDL-C: High-density lipoprotein cholesterol, LDL-C: Low-density lipoprotein cholesterol, Non-HDL-C: TC-HDL-C

### Anthropometric indices

Weight was measured with light clothes and without shoes using a digital scale (to the nearest 0.1 kg). Height was measured without shoes using a stadiometer (to the nearest 0.1 cm). Waist circumference was measured to the nearest 0.1 cm. BMI was calculated using the equation “BMI = weight/height^2^ (kg/meter^2^)”. The cut offs used in this study to characterize participants for obesity were based on the Executive Summary of the Third Report of the NCEP, ATP III and the IDF guidelines for South-Asian population [2, 17]. Obesity as a whole was defined as BMI >25 kg/m^2^; and central obesity was defined as WC >90 cm for males and WC >80 cm for females.

### Sample collection and handling

Blood samples were collected in fluoride, EDTA and serum vaccutainers between 8:00 to 11:00 am after 12 h of fasting for biochemical assays such as FPG, HbA1c, fasting insulin (FI), insulin resistance (HOMA-IR), and lipid profile estimation. Blood was collected again after 2 h of consuming non-standardized meal for PPPG estimation for T2DM subjects. Serum was separated within 30–45 min, aliquoted and stored at −20 °C till analysis.

### Biochemical investigations

FPG, PPPG and lipid profile including total cholesterol (TC), triglyceride (TG), and high-density lipoprotein cholesterol (HDL-C) were measured by colorimetric method with respective calibrator and biological standards. All biochemical investigations were carried out by commercially available kits using an auto-analyzer system (BTS 330, Biosystem, Spain). HbA1c was analyzed by affinity assay using the Nyco Card reader-II (Axis-Shield, Norway). FI levels were measured by Immuno Radiometric Assay (IRMA) with a commercial kit (Immunotech, France). Low-density lipoprotein cholesterol (LDL-C) and non-HDL-C were calculated by a standard formula [LDL = TC - HDL-C - (TG/5)] and [non-HDL-C = TC - HDL-C] [20]. Insulin resistance was calculated by the Homeostasis Model of Assessment-Insulin Resistance Index (HOMA-IR) with a model formula [HOMA-IR = (FI × FPG)/405] [21].

The intra assay coefficients of variations for lipid parameters were; TC: 1.39 %, TG: 0.50 % and HDL-C: 1.67 % whereas the inter assay coefficients of variations for aforesaid lipids were; 2.65 %, 1.65 % and 4.69 % respectively.

The cut offs for dyslipidemia used in this study were based on the IDF guidelines for South-Asian population. The hypercholesterolemia (hyper-TC) and hypertriglyceridemia (hyper-TG) were considered with TC ≥ 220 mg/dl and TG ≥ 150 mg/dl respectively; whereas, those with HDL-C ≤ 40 mg/dl, LDL-C ≥ 130 mg/dl and non-HDL-C ≥ 160 mg/dl were considered hypo-HDL-cholesterolemia (hypo-HDL-C), hyper-LDL-cholesterolemia (hyper-LDL-C) and hyper-non-HDL-cholesterolemia (hyper-non-HDL-C) respectively. TC < 220 mg/dl, TG < 150 mg/dl, HDL-C > 40 mg/dl LDL-C < 130 mg/dl and non-HDL-C < 160 mg/dl were regarded as normal in accordance with IDF and NCEP guidelines [2, 17]. Dyslipidemia is defined by presence of one or more abnormal serum lipid biomarker concentration.

### Statistical analysis

The sample size yielded a margin of error of 3.3 % in the study and a confidence limit of 95 % with an on-line calculator [22]. False positive error rate/Type I error was 0.05. The value of HbA1c was given as a percentage of total hemoglobin; and values of all other lipid parameters were given in mg/dl. All values were expressed as mean ± standard deviation. Linear regression, bivariate correlation analysis (using Pearson’s correlation coefficient) and student’s *t*-test analysis were carried out with IBM-SPSS v15.0. p values (two tailed) ≤0.05 were considered to be statistically significant.

## Results

### Assessment of Anthropometric indices and Biochemical parameters

Total 931 subjects were included in the study, 430 were T2DM (diagnosed for >6 months) and 501 were non-diabetic control subjects. Baseline characteristics of the participants are summarized in Table 1. The average duration of diabetes in T2DM subjects at inclusion was 8.39 ± 7.53 years (6 months to 40 years). Significantly higher mean FPG and HbA1c were observed in T2DM as compared to non-diabetic control subjects (FPG: p < 0.001 and HbA1c: p <0.001) respectively. Additionally, higher mean HOMA-IR, TC, TG and non-HDL-C were also observed in T2DM than in non-diabetic control subjects (HOMA-IR: p <0.001, TC: p = 0.008, TG: p < 0.001and non-HDL-C: p = 0.034) respectively.

### Prevalence of dyslipidemia and obesity

Dyslipidemia was seen in 50.27 % of the study population with highest in T2DM amongst both the groups followed by control subjects (53.72 % and 47.31 % respectively). Hyper-TC was observed in 19.76 % (85) and 20.56 % (103) of T2DM and control subjects respectively. Hyper-TG was higher in T2DM compared to control subjects [33.26 % (143) 19.56 % (98)], whereas hypo-HDL-C was 18.61 % (80) in T2DM and 17.54 % (88) in non-diabetic subjects. Furthermore, prevalence of hyper-LDL-C and hyper-non-HDL-C were almost at par in both the groups 23.02 % (99); vs. 25.95 % and 23.49 % (101) vs. 22.56 % (113) in T2DM compared to control subjects respectively (Data not shown).

The prevalence of central obesity was found to be higher (75 %) compared to peripheral obesity (59.83 %) in overall study population. Higher central obesity was seen in T2DM as compared to non-diabetic control subjects [82.56 % (355) vs. 70.26 % (352)] respectively. Similarly, incidence of peripheral obesity was also higher in T2DM than non-diabetic control subjects [66.27 % (283) vs. 54.29 % (272)] respectively (Data not shown).

### Association of dyslipidemia, central obesity and peripheral obesity with HbA1c levels

A significant linear association of hyper-TC, hyper-LDL-C and hyper-non-HDL-C with HbA1c was observed in T2DM and control subjects. Though, significant correlation between hyper-TG and hypo-HDL-C to HbA1c was not observed in subjects of the both groups (Table 2). Additionally, we also observed an association between normal HDL-C, LDL-C and non-HDL-C with HbA1c in control subjects. Though, higher WC or higher BMI per se had no independent association with HbA1c levels in any of the groups as shown in Table 2.

**Table 2:** Correlation of HbA1c on dyslipidemia and obesity

Parameters (T = 931 subjects)	T2DM (N = 430)			Controls (N = 501)		
	Association with HbA1c			Association with HbA1c		
	r^2^	r	p	r^2^	r	p
**Lipid parameters**						
**Hyper TC**	0.098	0.312	0.004*	0.030	0.172	0.003*
**Normal TC**	0.06	0.096	0.085	0.006	0.077	0.125
**Hyper TG**	0.001	0.036	0.670	0.000	0.010	0.921
**Normal TG**	0.002	0.043	0.483	0.000	0.019	0.705
**Hypo HDL**	0.000	−0.009	0.935	0.030	−0.174	0.106
**Normal HDL**	0.002	−0.050	0.369	0.012	−0.109	0.027*
**Hyper LDL**	0.101	0.317	0.001*	0.036	0.189	0.031*
**Normal LDL**	0.004	0.061	0.280	0.023	0.152	0.004*
**Hyper non-HDL-C**	0.137	0.370	<0.001*	0.050	0.224	0.017*
**Normal non-HDL-C**	0.001	0.030	0.598	0.031	0.176	0.001*
**Obesity**						
**Higher WC**	0.000	0.010	0.854	0.001	0.028	0.599
**Normal WC**	0.002	0.044	0.715	0.012	0.110	0.194
**Higher BMI**	0.000	0.009	0.887	0.012	0.110	0.071
**Normal BMI**	0.003	0.099	0.243	0.012	0.112	0.095

By Linear regression and bivariate analysis using Pearson’s correlation coefficient considering HbA1c as a dependent variable, *: Significant

BMI: Body mass index, WC: Waist circumference, TC: Total cholesterol, TG: Triglycerides, HDL-C: High-density lipoprotein cholesterol, LDL-C: Low-density lipoprotein cholesterol, non-HDL-C: TC-HDL

Hyper TC: TC ≥ 220 mg/dl, Hyper TG: TG ≥ 150 mg/dl respectively, Hypo-HDL: HDL-C ≤ 40 mg/dl, Hyper-LDL: LDL-C ≥ 130 mg/dl, Hyper-Non-HDL: Non-HDL-C ≥ 160 mg/dl, Higher WC: Male WC > 90 cm and Female WC > 80 cm, Higher BMI: BMI > 25 kg/m^2^

**Fig. 1:**
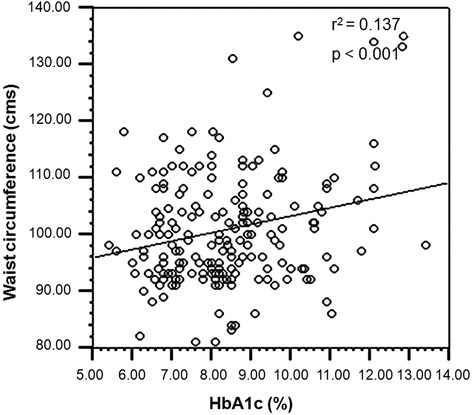
Association of central obesity with HbA1c in dyslipidemic T2DM subjects

**Fig. 2:**
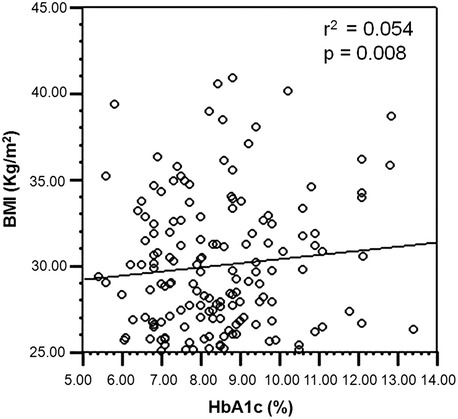
Association of peripheral obesity with HbA1c in dyslipidemic T2DM subjects

Nonetheless, significant linear association was observed in T2DM subjects with central- and peripheral- obesity along with dyslipidemia, while such correlation was not seen in non-diabetic subjects (Figs. 1 and 2).

## Discussion

The present population-based study was carried out on subjects from urban Western India with a mixed ethnicity. The major contributing factor for the higher risk of developing diabetes is likely to be due to increased prevalence of central- and peripheral- obesity in all the study subjects which is almost similar to the previous observation of ~30-65 % Indian urban being overweight or obese [23]. Obesity is known to be associated with increased amount of adipose tissue or its disproportionate distribution between central- and peripheral- body regions which is also related to the development of insulin resistance, dyslipidemia and CAD that increases the susceptibility of developing T2DM [24]. In addition to the storage of lipids in the adipose tissue, adipocytokines like adiponectin, leptin, TNF, IL-6, resistin play an important role in tissue physiology and have been shown to be linked to obesity, insulin resistance and β-cell dysfunction [24]. McTernan et al. in their animal study showed an increased resistin expression in adipose cells obtained from abdominal and omental region possibly contributing to insulin resistance, glucose intolerance and development of T2DM [25]. This could explain why obese T2DM subjects with dyslipidemia had poor hemoglobin glycation as compared to non-obese T2DM subjects in our study. The present study showed a higher number of T2DM subjects with central obesity as compared to peripheral obesity, which could be due to higher rates of lipolysis in visceral fat than subcutaneous fat by catecholamines [26]. This may in turn result into an increased FFA delivery to the liver, consequently stimulates hepatic glucose production by fatty acids (FA) causing interference in hepatic insulin removal, and may further accentuate insulin resistance [26].

Additionally, our subjects with hyper-TC, hyper-LDL-C and hyper-non-HDL-C alone or combined with obesity in T2DM group have shown poorly controlled HbA1c as compared to those with normal level of these parameters. Several reports have shown significant influence of lipid concentration on hemoglobin glycation and increased CVD risk possibly due to increased insulin resistance [27–29]. Nonetheless, these studies had stratified subjects based on their glycation control and observed their overall mean of lipid parameters, whereas in our study we had stratified subjects based on their dyslipidemia and obesity followed by observation of glycation pattern. Hyperglycemia promotes increase in LDL glycation and affinity towards LDL-receptors on macrophages; stimulate foam cell formation, endothelial cell toxicity and smooth muscle proliferation responsible for coronary artery and macrovascular complications [1].

We observed a significant association of non-HDL-C with HbA1c in our study which is in accordance with a recent study in Nepalese’ T2DM subjects [27]. Recent studies have also shown the utility of non-HDL-C as a significant biomarker for cardiovascular risk assessment and as a secondary target to monitor the effect of lipid lowering agents [3, 20]. It can be inferred that subjects with hyper-non-HDL-C are at an increased risk of CVD.

Our study could not demonstrate significant association of hyper-TG levels in T2DM subjects as compared to non-diabetic control subjects, contrary to the studies by Ikhas et al., and Schulze et al., which could be due to poor glycemic control that mask the effects of TG in higher glycation group [29, 30].

The Lipid Research clinics Coronary Primary Prevention Trial established that each 1 % fall in TC levels results into 2 % reduction in CAD risk [31]. A study carried out in Helsinki determined that with 11 % fall in LDL-C levels, there is 34 % reduced risk of CAD [31]. The present study observed an incidence of hyper-TC in 19.77 % of T2DM and 20.56 % of non-diabetic controls.

Moreover an increased dyslipidemia is likely to increase HbA1c and vice versa as the correlation between these parameters are directly proportional and goes hand-in-hand [27, 32]. Reduction in HbA1c in T2DM is associated with improved insulin sensitivity and better lipid parameters.

There are several mechanisms to elicit the effects of increased physical activity to improve dyslipidemia as it increases glucose removal and decreases muscle and hepatic IR through a number of mechanisms that would not necessarily be associated with changes in body weight [33]. This is in accordance with our results where we could not find correlation of HbA1c with BMI (which is an indicator of overall weight gain). However, increased physical activity and lifestyle modification seems to be associated with decreased HbA1c and better glycemic and lipid control. Thus, targeting to lower the dyslipidemia and obesity is likely to reduce HbA1c not only in diabetic subjects but it will have an equal effect in non-diabetic subjects.

## Conclusion

Dyslipidemia and obesity are significantly associated with poorly controlled hemoglobin glycation in T2DM and non-diabetic subjects. Additionally, higher prevalence of non-HDL-C in the study subjects suggesting its possible role as a biomarker for CVD.

## Abbreviations

T2DM: Type 2 diabetes mellitus
CVD: Cardiovascular disorders
HbA1c: Hemoglobin glycation
TC: Total cholesterol
TG: Triglyceride
HDL-C: High-density-lipoprotein cholesterol
LDL-C: Low-density-lipoprotein cholesterol
non-HDL-C: Non-High-density-lipoprotein cholesterol
CAD: Coronary artery disease
VLDL: Very-Low-density-lipoproteins
IDL: Intermediate- density-lipoproteins
NCEP-ATP III: National Cholesterol Education Program, Adult Treatment Panel III
WHO: World Health Organization
BMI: Body mass index
NEFA: Non-esterified fatty acids
IDF: International Diabetes Federation
ISBEC: Intersystem Biomedica Ethics Committee
WC: Waist circumference
FPG: Fasting plasma glucose
PPPG: Post prandial plasma glucose
FI: Fasting insulin
IRMA: Immune Radiometric Assay
HOMA-IR: Homeostasis Model of Assessment-Insulin Resistance Index
CHD: Coronary heart disease
DCCT: The Diabetes Complications and Control Trial
